# Supplementary material for: Resolving the full spectrum of human genome variation using Linked-Reads
Source: Genome Res. 2019 Apr;29(4):635–45. doi: 10.1101/gr.234443.118 (PMC6442396; doi:10.1101/gr.234443.118)
Supplement: Supplemental Material [file supp_gr.234443.118_Supplemental_longranger-2.2.zip › longranger-2.2.tar.bz2!/lr2.2-public/lib/stan/cmdstan/stan/doc/api/html/structstan_1_1agrad_1_1_operands_and_partials.html]

Stan: stan::agrad::OperandsAndPartials< T1, T2, T3, T4, T5, T6, T\_return\_type > Struct Template Reference


|  |  |
| --- | --- |
|  | Stan  2.5.0  probability, sampling & optimization |


- Main Page
- Related Pages
- Namespaces
- Classes
- Files

- Class List
- Class Index
- Class Hierarchy
- Class Members

All Classes Namespaces Files Functions Variables Typedefs Enumerations Enumerator Friends Macros Pages

- stan
- agrad
- OperandsAndPartials

Public Member Functions |
Public Attributes |
Static Public Attributes |
List of all members

stan::agrad::OperandsAndPartials< T1, T2, T3, T4, T5, T6, T\_return\_type > Struct Template Reference

A variable implementation that stores operands and derivatives with respect to the variable.
More...

`#include <partials_vari.hpp>`

|  |  |
| --- | --- |
| Public Member Functions | |
|  | OperandsAndPartials (const T1 &x1=0, const T2 &x2=0, const T3 &x3=0, const T4 &x4=0, const T5 &x5=0, const T6 &x6=0) |
|  | |
| T\_return\_type | to\_var (double logp) |
|  | |

|  |  |
| --- | --- |
| Public Attributes | |
| size\_t | nvaris |
|  | |
| agrad::vari \*\* | all\_varis |
|  | |
| double \* | all\_partials |
|  | |
| VectorView< double   \*, is\_vector< T1 >::value,   is\_constant\_struct< T1 >  ::value > | d\_x1 |
|  | |
| VectorView< double   \*, is\_vector< T2 >::value,   is\_constant\_struct< T2 >  ::value > | d\_x2 |
|  | |
| VectorView< double   \*, is\_vector< T3 >::value,   is\_constant\_struct< T3 >  ::value > | d\_x3 |
|  | |
| VectorView< double   \*, is\_vector< T4 >::value,   is\_constant\_struct< T4 >  ::value > | d\_x4 |
|  | |
| VectorView< double   \*, is\_vector< T5 >::value,   is\_constant\_struct< T5 >  ::value > | d\_x5 |
|  | |
| VectorView< double   \*, is\_vector< T6 >::value,   is\_constant\_struct< T6 >  ::value > | d\_x6 |
|  | |

|  |  |
| --- | --- |
| Static Public Attributes | |
| static const bool | all\_constant = is\_constant<T\_return\_type>::value |
|  | |

## Detailed Description

### template<typename T1 = double, typename T2 = double, typename T3 = double, typename T4 = double, typename T5 = double, typename T6 = double, typename T\_return\_type = typename return\_type<T1,T2,T3,T4,T5,T6>::type> struct stan::agrad::OperandsAndPartials< T1, T2, T3, T4, T5, T6, T\_return\_type >

A variable implementation that stores operands and derivatives with respect to the variable.

Definition at line 76 of file partials\_vari.hpp.

## Constructor & Destructor Documentation

template<typename T1 = double, typename T2 = double, typename T3 = double, typename T4 = double, typename T5 = double, typename T6 = double, typename T\_return\_type = typename return\_type<T1,T2,T3,T4,T5,T6>::type>

|  |  |  |  |
| --- | --- | --- | --- |
| stan::agrad::OperandsAndPartials< T1, T2, T3, T4, T5, T6, T\_return\_type >::OperandsAndPartials | ( | const T1 & | *x1* = `0`, |
|  |  | const T2 & | *x2* = `0`, |
|  |  | const T3 & | *x3* = `0`, |
|  |  | const T4 & | *x4* = `0`, |
|  |  | const T5 & | *x5* = `0`, |
|  |  | const T6 & | *x6* = `0` |
|  | ) |  |  |

inline

Definition at line 89 of file partials\_vari.hpp.

## Member Function Documentation

template<typename T1 = double, typename T2 = double, typename T3 = double, typename T4 = double, typename T5 = double, typename T6 = double, typename T\_return\_type = typename return\_type<T1,T2,T3,T4,T5,T6>::type>

|  |  |  |  |  |  |
| --- | --- | --- | --- | --- | --- |
| T\_return\_type stan::agrad::OperandsAndPartials< T1, T2, T3, T4, T5, T6, T\_return\_type >::to\_var | ( | double | *logp* | ) |  |

inline

Definition at line 138 of file partials\_vari.hpp.

## Member Data Documentation

template<typename T1 = double, typename T2 = double, typename T3 = double, typename T4 = double, typename T5 = double, typename T6 = double, typename T\_return\_type = typename return\_type<T1,T2,T3,T4,T5,T6>::type>

const bool stan::agrad::OperandsAndPartials< T1, T2, T3, T4, T5, T6, T\_return\_type >::all\_constant = is\_constant<T\_return\_type>::value

static

Definition at line 77 of file partials\_vari.hpp.

template<typename T1 = double, typename T2 = double, typename T3 = double, typename T4 = double, typename T5 = double, typename T6 = double, typename T\_return\_type = typename return\_type<T1,T2,T3,T4,T5,T6>::type>

double\* stan::agrad::OperandsAndPartials< T1, T2, T3, T4, T5, T6, T\_return\_type >::all\_partials

Definition at line 80 of file partials\_vari.hpp.

template<typename T1 = double, typename T2 = double, typename T3 = double, typename T4 = double, typename T5 = double, typename T6 = double, typename T\_return\_type = typename return\_type<T1,T2,T3,T4,T5,T6>::type>

agrad::vari\*\* stan::agrad::OperandsAndPartials< T1, T2, T3, T4, T5, T6, T\_return\_type >::all\_varis

Definition at line 79 of file partials\_vari.hpp.

template<typename T1 = double, typename T2 = double, typename T3 = double, typename T4 = double, typename T5 = double, typename T6 = double, typename T\_return\_type = typename return\_type<T1,T2,T3,T4,T5,T6>::type>

VectorView<double\*, is\_vector<T1>::value, is\_constant\_struct<T1>::value> stan::agrad::OperandsAndPartials< T1, T2, T3, T4, T5, T6, T\_return\_type >::d\_x1

Definition at line 82 of file partials\_vari.hpp.

template<typename T1 = double, typename T2 = double, typename T3 = double, typename T4 = double, typename T5 = double, typename T6 = double, typename T\_return\_type = typename return\_type<T1,T2,T3,T4,T5,T6>::type>

VectorView<double\*, is\_vector<T2>::value, is\_constant\_struct<T2>::value> stan::agrad::OperandsAndPartials< T1, T2, T3, T4, T5, T6, T\_return\_type >::d\_x2

Definition at line 83 of file partials\_vari.hpp.

template<typename T1 = double, typename T2 = double, typename T3 = double, typename T4 = double, typename T5 = double, typename T6 = double, typename T\_return\_type = typename return\_type<T1,T2,T3,T4,T5,T6>::type>

VectorView<double\*, is\_vector<T3>::value, is\_constant\_struct<T3>::value> stan::agrad::OperandsAndPartials< T1, T2, T3, T4, T5, T6, T\_return\_type >::d\_x3

Definition at line 84 of file partials\_vari.hpp.

template<typename T1 = double, typename T2 = double, typename T3 = double, typename T4 = double, typename T5 = double, typename T6 = double, typename T\_return\_type = typename return\_type<T1,T2,T3,T4,T5,T6>::type>

VectorView<double\*, is\_vector<T4>::value, is\_constant\_struct<T4>::value> stan::agrad::OperandsAndPartials< T1, T2, T3, T4, T5, T6, T\_return\_type >::d\_x4

Definition at line 85 of file partials\_vari.hpp.

template<typename T1 = double, typename T2 = double, typename T3 = double, typename T4 = double, typename T5 = double, typename T6 = double, typename T\_return\_type = typename return\_type<T1,T2,T3,T4,T5,T6>::type>

VectorView<double\*, is\_vector<T5>::value, is\_constant\_struct<T5>::value> stan::agrad::OperandsAndPartials< T1, T2, T3, T4, T5, T6, T\_return\_type >::d\_x5

Definition at line 86 of file partials\_vari.hpp.

template<typename T1 = double, typename T2 = double, typename T3 = double, typename T4 = double, typename T5 = double, typename T6 = double, typename T\_return\_type = typename return\_type<T1,T2,T3,T4,T5,T6>::type>

VectorView<double\*, is\_vector<T6>::value, is\_constant\_struct<T6>::value> stan::agrad::OperandsAndPartials< T1, T2, T3, T4, T5, T6, T\_return\_type >::d\_x6

Definition at line 87 of file partials\_vari.hpp.

template<typename T1 = double, typename T2 = double, typename T3 = double, typename T4 = double, typename T5 = double, typename T6 = double, typename T\_return\_type = typename return\_type<T1,T2,T3,T4,T5,T6>::type>

size\_t stan::agrad::OperandsAndPartials< T1, T2, T3, T4, T5, T6, T\_return\_type >::nvaris

Definition at line 78 of file partials\_vari.hpp.

---

The documentation for this struct was generated from the following file:

- src/stan/agrad/partials\_vari.hpp


---

[ Stan Home Page ]

*© 2011–2014,
Stan Development Team.*
